# Platforms of Work, Labour, and Employment Relationship: The Grey Zones of a Digital Governance

**DOI:** 10.3389/fsoc.2020.00002

**Published:** 2020-02-19

**Authors:** Patrick Dieuaide, Christian Azaïs

**Affiliations:** ^1^Université de la Sorbonne Nouvelle, Paris, France; ^2^Conservatoire National des Arts et Métiers (CNAM), Paris, France

**Keywords:** digital governance, grey zones, employment relationship, nudges, labour

## Abstract

Drawing on numerous case studies, the article examines the specific conditions for organising and managing the employment relationship on digital labour platforms. We show that these conditions are largely due to the disruptive nature of the process of digitising the employee–employer relationship. Digitisation replaces the employment contract of the standard employment relationship with a triangular “worker–platform–customer” relationship. In this model, the boundaries of the employment relationship become opaque and more uncertain: the bond of subordination disappears, labour law gives way to commercial law, and the figures of the employer and the employee lose institutional visibility. The article seeks to clarify the contours of this “in-between” model and proposes the notion of the “grey zone,” borrowed from geopolitics. This notion of the “employment grey zone” makes it possible to shift the researcher's perspective by focusing attention on practices and “intermediate spaces of regulation,” which are relatively autonomous and endowed with their own dynamics. This framework of analysis broadens the perspective and helps to better understand the impact on the employment relationship of new forms of governance in a context of a digital turning point. The article first returns to the notion of the “grey zone” and argues on the foundations and interest of mobilising this notion in the field of industrial relations studies. The links between digital platforms and grey zones are then examined. In particular, we show that digital governance is based on a confusion of powers between coordination and leadership. The reflection continues in a third phase with an examination of digital management practices in two areas: the control of the activity of connected workers, and the production and management of externalities resulting from the operation of platforms. The article concludes with a discussion on the heuristic value of the notion of grey zones of employment.

## Digital Platforms And Employment Governance: Introductive Issues

We are all familiar with the multinational company Uber and its legal wrangling with professional taxi drivers, and also with the conflict between Airbnb and the hotel industry. Uber experimented with an original business model based on bringing together customers and connected workers who have their own private car, a driving licence, a transport network company (TNC) drivers licence (for a chauffeur-driven car), and professional insurance; the premise behind Airbnb is to make private apartments available, renting them to customers, usually tourists, for a short time only. In both cases, neither the drivers' labour power nor the different types of capital involved belong to these companies. Uber owns no cars, and the workers who drive them are legally independent contractors; Airbnb, similarly, owns no accommodation; these two American giants are content to be merely intermediaries in the market.

With no real assets and a minimum number of permanent employees and allocating most of their budget to developing search engines and marketing (Acquier, 2017), digital platforms are companies that are also difficult to define in legal, institutional, and fiscal terms. For example, Uber-France is classified by statisticians at National Institute of Statistics and Economic Studies (INSEE) in the national classification of activities under code APE 8299Z (“other business support activities”), in fact in the similarly vague subset “enterprises not classified elsewhere,” a heading that does not correspond to any of these businesses' known activities (catering service and mobility service). What is Uber's activity? Is Uber a service enterprise or a technology enterprise as its directors claim? Or is it a transport company, as it was recently described by the judges at the European Court of Justice? Similarly, one can wonder about the founding principles of the business model of this company, which after 8 years in business continues to record a loss, which declares a turnover of only 52 million euros (compared with an estimated turnover of 240 million for the G7 taxi company) and which, thanks to tax optimisation, pays the French state a derisory 1.4 million euros in taxes. More generally, and although not all platforms have such a high profile as Uber, the digitisation and transformation of these enterprises into hollow corporations enable them to free themselves from many legal and regulatory frameworks, whether in competition law, labour law, or tax law.

In addition, the activity of digital platforms like Uber's has its foundation in a very real technological and social base, with firm local attachments. Equipped with a data centre, smartphones, and an application (algorithm), their platforms underpin vast networks of local social relations. Players and/or activities are scattered geographically but are brought together, and among themselves, they create many market transactions on the basis of which these companies receive remuneration by charging a commission. From a management point of view, this role as market intermediary, with their ear as close to the ground as possible, is the provision of information services. Their aim is to facilitate exchanges by ensuring the quality of matches between the different platform users, by perfecting the algorithms in order to meet expectations better, and by guaranteeing the smooth running of transactions (Tirole, 2018).

**Figure 1:**
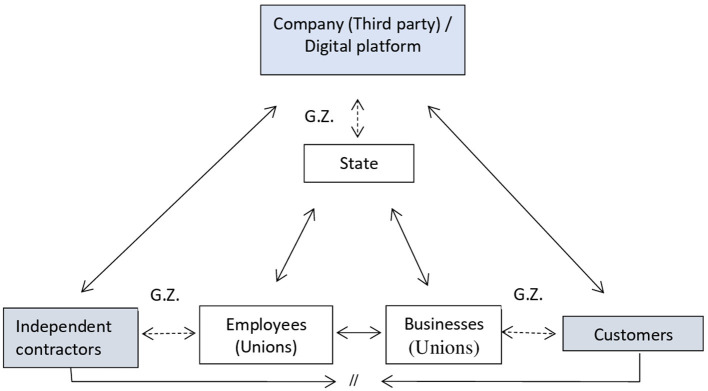
Industrial relations, digital platform, and triangulation of the employment relationship.

In their role as market intermediary, digital platforms have proliferated in a growing number of sectors, and as a result, the scope of the triangulation principle (Dieuaide, 2018) has been widened considerably. At the boundaries between professional relations and the employment relationship, an alternative model is emerging, consisting of services between customers (suppliers) and independent contractors *via* a company (or a third party) that is largely autonomous vis-à-vis existing institutional frameworks (see Figure 1).

Based on the “click” economy (Casilli, 2019), digital labour platforms have been repeatedly denounced as the gravediggers of wage labour (Abdelnour, 2017). On the one hand, they encourage the outsourcing of business and corporate jobs (Drahokoupil and Fabo, 2016); on the other hand, they increase competition in the labour market and lead to a sharp deterioration of wages and working conditions (Eurofound, 2018).

However, we believe that many of these approaches underestimate the disruptive effects associated with the widespread dissemination of digital technologies (Wajcman, 2006). Digital platforms are not companies “like any other.” By working on data collected from the internet community, platforms not only act as third party mediators for the production of informational services but also behave like prescribers in that disseminated information enters directly into the agents' decision-making processes. Platforms are not only technological devices but also psychologically relevant entities (Carolus et al., 2018, p. 21). As many surveys have shown, this prescriptive power is a source of stress and addiction for the workers (Huws et al., 2016). Whether intentionally or not, it is therefore a source of confusion: in addition to being a service relationship, it is a relationship of influence that independent workers and customers must endure and in which they have no way of intervening. In other words, platform workers are neither completely independent nor completely subordinate. Similarly, platform companies are not quite market intermediaries, nor quite employers.

How do we characterise the employment relationship in such a context? Can we still talk about an employment relationship when the employer is nothing more than a matching algorithm? What autonomy and what work are we talking about in the context of an employment relationship that is governed digitally?

To answer these questions, it is not enough to invoke deviations from the standard employment norms or to point out the existence of non-law zones. The employment relationship attached to labour platforms cannot be reduced to disorder or even institutional chaos. It is more of a social, political, and historical construct, based on hybrid discourses and practices, neither too visible, nor too invisible, neither legal, nor illegal. As mentioned above, the terms “self-employed,” “employers,” and even “clients” are not self-evident (Eurofound, 2017). Behind each of them lies an ambivalent and complex reality, and it would be a very unsatisfactory method if we were to transpose the traditional analytical frameworks from industrial relations studies to reveal all their facets.

This ambivalence and complexity of the employment relationship require researchers to shift their focus. To do this, we propose to use the notion of the “grey zone,” a notion that comes from geopolitics and that, when imported into the field of industrial relations studies, offers the advantage of a better contextualisation of our research object and thus leads to new questions and new perspectives for analysis.

The approach we propose takes up and extends the discussion in a number of studies on the subject (Transfer, 2018). With some of the usual precautions, to which we will return later, the term “grey zone” makes it possible to draft a framework for interpreting transformations in the employment relationship on the basis of the observation of a divergence between institutions and the behaviour and practices of the actors. More precisely, two possible interpretations of the notion of grey zone emerge from these studies: a first reading equates the grey zone of employment with a loss of effectiveness of existing institutions and/or legal instruments; a second reading considers the notion of the grey zone of employment as the expression of a “non-standard” regulation, that is, a regulation implemented and/or directly carried out by actors or a community of actors unofficial who act or behave “without” or “outside” the rules.

As part of this contribution, we intend to take this framework of analysis and use it to decipher the specific terms of organisation and management of the employment relationship in these unprecedented productive worlds, commonly known as “capitalism platforms^1^.”

The interest of this approach is to open up discussion on the transformations of the employment relationship by paying particular attention to the new forms of governance that have emerged with the development of digital information management and processing technologies. This viewpoint will lead to questions about the impact of this dematerialisation process on the organisation and implementation of the management power held by the owner-managers of the labour platforms.

This reflection will be in three stages. In the first part, we will present our general framework for analysing employment grey zones. We will specify the terms of the rupture brought about by the “digital turn” (Valenduc, 2019) by insisting on two closely related disruptive effects: the rejection or negation of the standard employment relationship on the one hand, and the recognition of the notion of the grey zone of employment as an “intermediate space of regulation” on the other. In the second part, we discuss the close and ambivalent link between platforms and grey zones. First, we clarify the foundations of this dual structure of the power held by the platform managers, both a power to connect and a power to direct. Second, we draw up a typology of grey zones. In the third part, we examine the impact of digitisation on management practices. Based on numerous case studies, two key areas will be addressed: monitoring the activity of connected workers, and the production and management of externalities. The article concludes with an overview and a discussion of the heuristic interest of the notion of an employment grey zone.

## A General Framework for Analyzing Employment Grey Zones

“Capitalism of surveillance” (Zuboff, 2019), “cognitive capitalism” (Boutang, 2012), “platform capitalism” (Srnicek, 2017): the proliferation of terms betrays the difficulty of grasping contemporary mutations linked to digital technology diffusion. The following developments are part of the continuity of this debate. We will question why platform leaders have always refused to consider themselves as employers and therefore recognise connected workers as employees.

### Rejection of the Standard Employment Relationship in Platform Capitalism

To understand the close relationship between digital platforms and grey zones, it is worthwhile to first remind ourselves of Marx's conception of the labour process, as developed in Volume 1 of *Capital*. For Marx, the labour process is a combination of several components: the worker's labour power on the one hand and intermediate consumption and the means of production used or consumed on the other hand, giving the result or product of labour. For Marx, these different components are the property of capitalists. All the more so because the way in which these components are organised and the way in which the products of labour are designed and distributed on the goods market mean that they are placed directly under its responsibility and control. In this approach, the employment relationship is the hub of the capitalist business: it represents this specific moment when the worker's labour power, negotiated and sold to “moneybags” for a given time, is consumed in a productive fashion before being remunerated; this relationship then makes wages (and wage earners) the keystone of social relations of domination as well as being an essential condition to reproduce the workers' living conditions (Lautier and Tortajada, 1978).

In contrast, since the end of the 2000s, businesses like Uber (2009) and Deliveroo (2013) have emerged, whose productive characteristics are the complete antithesis of this “labour” model of the employment relationship. These multinationals present themselves as businesses with no factories and who are backed massively by risk capital; they produce nothing directly for the goods and services market and pay no wages to their thousands of connected crowdworkers. In other words, at first glance, there is nothing in the characteristics of these market intermediaries that suggests the slightest hint of direct involvement by its managers in the organisation. On the AMT type of work platform, these companies provide no explicit work goals; they set no tasks and assign no place in any organisation whatsoever. In short, and with all due respect to Marx, platform capitalism appears to be embodied in a business model that is virtually empty of any social form of employment or labour relationship.

On the other hand, as Benavent points out (Benavent, 2016, p. 86), digital platforms are very powerful tools for networking and coordination and have no boundaries in space or time. Platform managers will know perfectly well how to derive benefit from this characteristic, in that the digitisation of “productive meetings” organised and managed by the platforms need have no regard for the general and concrete conditions of organising the activities and operation of the markets. The result is a radical reversal of perspective: in exercising their power of coordination, managers no longer need to be backed locally by private ownership of the human and material components of the labour process, nor is it even necessary to draw up a contract of employment setting out the conditions of use and remuneration for the worker's labour power. Through digitisation, the platforms control and manage remotely the information base that governs the organisation and management of labour relations at the local level. As Serrano-Pascual and Jepsen point out (2018, ch. 14), the employment relationship has become a notion whose meaning is at stake in a semantic and political battle between different social groups.

There is therefore no need, in principle, to create value as a stakeholder in community governance embedded locally in the organisational (Havard et al., 2006) and institutional framework of a company in a given country. In platform capitalism, the institutions and collective social rights that make up the employment relationship and wage relations, in general, are literally subsumed by digitisation and the network rationale. With digitisation, work is perceived by management as supply and demand for services. In other words, the reason these institutions were created in the time of Fordism no longer has a place in this new configuration. Legal protection and social rights attached to the workers' person are no longer guaranteed. In the digital world of platforms, the standard employment relationship is no longer the norm (Brishen, 2016), apart from appearing in a negative way, either by putting up a legal obstacle to connecting platform users or as examples of institutions that are expensive to run and not compatible with the principles of a business model founded on flexibility (De Stefano, 2018) and on collecting, processing, and disseminating information to the greatest number of platform workers.

### The Notion of “Employment Grey Zone” as an Intermediate Space of Regulation: The Contribution of Geopolitics to Analysis

From the preceding reflection, it emerges that digital platform managers are not keen to take on the role of employer, even if, by processing and using the information they collect, these same managers can sometimes act as managers or at least behave as if they were, if unwittingly (Cardon, 2019). The many appeals to the courts by Uber drivers to convert service contracts into employment contracts in France, the United States, England, and elsewhere are an illustration of this^2^.

“Being an employer” or “behaving like one”: the nuance may go unnoticed but it is key to the analysis. This highlights the extreme vagueness surrounding the responsibilities incumbent on those who manage work platforms. More fundamentally, it demonstrates the existence of a legal “no man's land” where managers' actions can sometimes slip from a power of matching (or coordinating), which is essentially global or transverse, to a power to direct, with a local or limited dimension.

In a work devoted to the notion of grey zones, the political scientist G. Minassian describes this confusion of genres as a “symptom of social pathologies in the world space” (Minassian, 2018, p. 22) and proposes a definition of the notion of grey zone that is very relevant for our purpose. For Minassian, a grey zone is:

“a space—with or without a fence—of social deregulation, of a political nature (self- determination, separatism or sanctuarisation) or socioeconomic nature (criminality spaces, dehumanised spaces, desocialised spaces), essentially terrestrial, sometimes maritime, dependent on a sovereign State whose central institutions are unable (either through powerlessness or abandonment) to penetrate it in order to assert their domination, which is ensured by alternative micro-authorities” (Minassian, 2018, p. 16).

From this long definition, Minassian draws three essential principles that characterise a grey zone (Chapter 2, we summarise):

- a principle of competition with authority where the state is openly challenged in its role of keeper of the peace and in its capacity to ensure the safety and protection of its people;
- a principle of social deregulation that reflects a lack of social contract between the state and society and manifests itself in a certain number of social pathologies (unemployment, recession, poverty, etc.) and a deterioration in social relations (violence, incivility, and rise in communitarianism);
- a principle of privatisation of the territory, driven on the one hand by the arrival of huge numbers of transnational players (multinational firms, financial capital, non-governmental organisations NGOs, and social networks) and on the other by the many locally based interest groups and defenders of particularistic and traditional values.

By analogy, we propose to define the grey zone in the field of employment and labour relations as an intermediate space of regulation, closely linked to the development of digital platforms, a space that we characterise according to Minassian's three principles:

- The principle of competition with authority refers to the power of platforms to choose freely the place where their company headquarters are located. The ubiquity of computer systems makes it possible not only to escape the payment of taxes (O'Keeffe and Jones, 2015) but also to avoid, to a large extent, any obligations under the Labour Code, as in the case of France. Thus, the employer of Uber drivers working in France, Uber BV, is domiciled in the Netherlands. For the lawyer A. Supiot, freedom of choice masks a practice known as “law shopping” (Supiot, 2010), which in fact pits national legislations against each other and places pressure on national parliaments in favour of social dumping.
- The principle of social deregulation refers to various legal loopholes, either because the labour law currently in force is not applicable or because, quite simply, it is not applied because such law does not exist: these are all scenarios that have been observed with the arrival of these businesses in cities, especially in the mobility and catering service sectors (Uber, Lime, Deliveroo, etc.). Note that this lack of protection is found in highly standardised professions such as helicopter pilot (Azaïs, 2019a), a sure sign that the grey zone affects all types of profession, from the most “traditional” (i.e., closer to the Fordist regulatory norm) to the newest. The expression “the uberisation of jobs” gives a fairly good idea in everyday language of the phenomenon of deinstitutionalising the employment relationship or distancing platform workers from wage earners' institutions (unemployment insurance, collective bargaining, and recognition of rights and status associated with employee status).
-The principle of privatisation (or appropriation) of the territory is based on mobilising all available social wealth on which the platforms rely in order to operate. This may be urban and rural road infrastructure (as in the case of Uber), residential buildings (Airbnb), business premises (restaurants), and more broadly, any use value (bicycle, boat, private car, helicopter, etc.) and any available person who may be digitally connected (or interrelated). As they are firmly anchored locally, digital platforms perform their activity of matching people at the same time as they occupy the public space, dividing it up, and, depending on the type of activity, exploiting the productive and creative potential. In short, it is as if the platforms perceive local territories not only as resources distributed in an open space, free to access, but also as potential markets that they compete for control over *via* digitisation (Ashton et al., 2017). Embedded in their digital networks, territories are an inexhaustible reservoir of data that the platforms collect, transform, and disseminate to their members as usable information. In this operation, the economic and commercial interests of the platforms do not necessarily match those driven by the local areas, which are institutional players and spaces regulated according to more general and collective norms or interests. This divergence accounts for the appearance of tensions that has resulted in some municipalities banning Uber from operating in their territory: among them Barcelona, Frankfurt, Rome, or Sofia. From some municipalities, such as Austin (Texas), both Uber and Lyft companies have left not wanting to meet the fingerprint requirement, in 2016. They came back in 2017, because the regulations were more flexible.

In Rio de Janeiro, for example, the municipality is proposing the coupling of the transport pass Giro with Uber, offering a 30% discount on the price displayed on the meter for the route made with Uber.

However, other *scenarii* are possible, such as in Dublin, California, where Uber has negotiated a partnership that allows them to cover sections of their urban territory that are poorly served by public transport but stipulates that they apply a single price of US $5 for any fare within their boundaries. But more often than not, local authorities are very suspicious of platforms. For example, the City of Paris has decided to sue Airbnb for illegal advertising of rentals on its site (Serafini, 2019). For Cannon and Summers (2014), more dialogue would bring many benefits in terms of job creation, tax revenue, attractiveness, or services offered to users or consumers. These retaliatory measures against the platforms are simply a sign of a reaction against a power perceived as invasive and disrupts the socio-political balances that previously existed.

In the field of employment and labour relations, this becomes clear: in the world of the digital workplace, employment is no longer part of an employee–employer face-to-face relationship but takes place in an open, transnational, public and private, local and global space. In this grey zone, at different levels and at different times, there is a diversity of stakeholders involved in a plural and complex regulation (Azaïs and Pepin-Lehalleur, 2014). In the words of the sociologist J.D. Reynaud, the employment grey zone is based on the existence of a plurality of sources of regulation (Reynaud, 2003).

As we have observed in France in the Uber case (Azaïs et al., 2017), regulation becomes the issue and the stage for consultations and often for disputes involving a diverse collection of public entities (platform users, local authorities, professional associations, civil society, government, etc.), depending on the questions asked (training of drivers, competition with taxis, safety, use of the public space, etc.) and the interests that are challenged. In France, regulation is therefore multilevel, mediated by the state and to a large extent focused on maintaining a balance with the taxi driver profession in the discord that has gradually emerged with the arrival of Uber in the mobility market. However, the comparative study conducted by Thelen also shows that countries did not all react in the same way to the Uber shock. In the United States, Uber has established itself by forging an alliance with consumers against unpopular taxi lobbies, albeit at the cost of long legal battles with the lawyers of the workers connected to the platform. In contrast, in Germany, the alliance of taxis with public transport professionals, in the name of defending a high-quality and reliable service, has resulted in the closure of market access (except Berlin and Munich). Similarly, in Sweden, a broad coalition of taxi companies, trade unions, and state actors lobbied to tax Uber's activity in order to defend the equity standards on which the Swedish social system is based (Thelen, 2018). In Brazil, the municipality of Rio de Janeiro decided to lower the taxi drivers' fare to compete with Uber's fares. To do so, it created an app, app.taxi.rio. The meter price is systematically reduced by 30%.

In this context, regarding regulation, the “ability to make rules can [therefore] be characterised by the place in an interaction for those whose initiative it is” (Reynaud, 2003, p. 103), so there is every chance that governance of the employment relationship will come up against too many forms of mediation and will always appear partial and unable to contain the conflicts and dynamics that are operating.

In short, the employment grey zone appears as a place of concentration of micropowers. In this multifaceted space, work platforms and the employment grey zone go hand in hand. The former feed on the latter, which in turn tends to continue and prosper with the development of the former. This codetermination has the effect of crystallising well-understood interests, consolidating acquired positions, and possibly sealing relatively stable compromises, which may or may not last. This dynamic should not be underestimated. It carries with it the springs of its own development to the point of imposing itself as an essential cog in the mode of operation of the platforms.

## Between Digital Platforms and Grey Zone: Close and Ambivalent Links

Reduced to their basic function, digital platforms are information processors. As Srnicek emphasises, platforms are central models for extracting data as raw material to be used in various ways (Srnicek, 2017, p. 45). They collect data and transform them into usable information for the connected users. But this activity is also the moment when they take control of these data in the absence of an exchange^3^. This control is usually framed in a legal context in a document displayed on the platform websites and spells out for the users the terms and conditions for using these data^4^. However, this limitation is usually ill-founded because the distinction between collected data and new data produced and transmitted by the platform to users is very unclear. How is this new concept defined? There is a grey zone here that is pushing against the boundaries of the platforms' freedom to use these data in the way they want.

### Dualism and the Power of Prescription of Digital Platforms

In general, the economic and sociological literature considers digital platforms as market operators (Tirole, *op. cit*., Cardon, *op. cit*.). By facilitating meetings between suppliers and customers of goods and services, they increase market effectiveness and improve the level of utility or well-being of its users. In this way, platforms have a regulatory power by carrying out an entire series of actions, for example (Tirole, *op. cit*.):

- They host many applications and ensure a degree of competition between businesses.
- They regulate prices by imposing maximum price levels, and they protect consumers by monitoring contents and behaviours.
- They monitor the quality of services offered (dating agencies and standards at Uber).
- They check the reliability of sellers (examine drivers' background) and arbitrate disputes (deactivate drivers).

This service-based approach to the activity of the platforms is only partial and even restrictive as it overlooks the conditions under which the platforms intervene in the setting up the organisation and the management of the markets. As Gauron points out, the innovation that the platforms introduced “lies in the fact that they have replaced a direct relationship between individuals and they have killed the free aspect and the solidarity that oversaw this relationship when it had existed” (Gauron, 2017).

This comment adds a new and important element to the analysis of the grey zones carried out earlier. It shows that the purpose of the matching process on the platforms was not only to facilitate relations between suppliers and customers, in exchange for payment, especially by classifying, filtering, and ranking information disseminated to users; this process is also based on a technical mechanism to create a relationship that establishes a market trade link between users and platforms. In the result, the relationship between the customer-supplier and the service provider is duplicated and any possibility of direct communication between them becomes impossible (see diagram in Figure 1).

**Figure 2:**
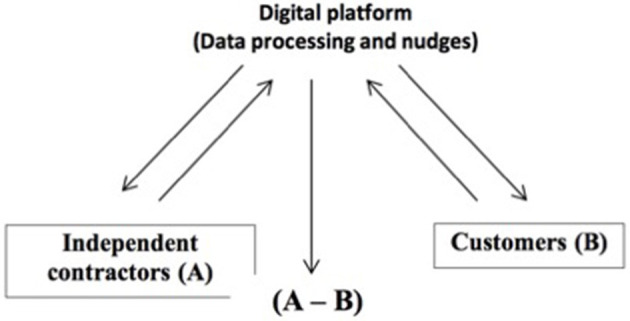
Digital platforms as the intermediary of information management.

Consequently, the creation of markets by the platforms imposes not one but two levels of constraint on users: the first level is accepting the digital format as the only possible way of entering into the relationship; the second level is accepting that, on this basis, the platforms have direct and exclusive access to the digitised data that is transmitted upstream to organise and manage these relations. In these circumstances, the activity of the platforms is identical in many ways to the work carried out by the Gosplan office^5^: platforms are permanently fed data transmitted by the community of users, operating on the one hand like an information system that is infinitely (re)programmable (*via* the algorithms) while on the other hand imposing itself as a means of communication, organising and directing users' behaviours and choices (see Figure 2).

This dual structure breaks from the principle of neutrality generally attached to the role of market intermediary. In return, it gives platforms the opportunity to govern markets that they are able to create from scratch (!). As direct and exclusive interlocutors of A and B (see Figure 2), platforms are active and in a monopoly position on both sides of the market (Rochet and Tirole, 2003). This position provides them a power of influence that allows discretionary management of exchanges between A and B, taken directly from the information that guides the choices and behaviours of each. If we take the case of Uber, discretionary management is confirmed, among other things, by the platform's power to set the price of the ride and to change the percentage of commission the driver receives unilaterally, also by its power to deactivate drivers whose customer ratings are not high enough (Birgillito and Birgillito, 2018).

### An Attempt at a Typology of Grey Zones

Consequently, from the point of view of employment and labour relations, the way that digital platforms operate highlights a radical transformation in the governance mechanism that coordinates and carries out the activities of connected workers.

Whereas, in the standard employment relationship these activities were supervised under the direct and contractual responsibility of the employer, the governance of these activities in the case of platforms is no longer legally regulated. The employee–employer relationship, built on a common desire and the reciprocity of the parties' commitments to the employment contract, is replaced by a service relationship with no obligations in terms of social protection and working conditions. On platforms like those of Uber or Deliveroo, governance is limited to an information system that has at its heart a price fixing algorithm coupled with incentive schemes or sanctions (deactivation), surveillance (geolocation), and rating of the service provided (delegated to the customers in the case of Uber). In such a context, three types of grey zone can be identified.

The first type relates to the nature and extent of the coordination or matching power wielded by the platform managers. Where does this matching power begin and end? This is a very discerning question to ask, because in the case of Uber and Deliveroo, the fact of making a digital connection, which is necessary in order to open an account, is equivalent to “self-declaration:” the connected worker makes a quasi-unilateral commitment (Aloisi, 2016). By signing up on the platform and becoming a member, he becomes an independent contractor—this status is the *sine qua non* condition for being able to carry out one's future activity—and declares his availability to provide the service at any time. This matching of driver and customer is therefore not symmetrical but asymmetrical (Kingsley et al., 2015): this is a direct consequence of the platform's taking over the customer relationship, which becomes a relationship of economic dependency with the worker dependent on the platform. Thus, the information sent to the drivers is the equivalent of an order (no matter how small the value of this order may be) and the platform acts implicitly as the ordering party. Between the platform and the connected workers, there is the same kind of relationship as that between a large company and its suppliers or subcontractors. The boundary between coordinating power and management power is therefore a tenuous one. It stems only from the leaders' management style or more broadly from the degree of external economic pressure such as competition or the profitability requirements of the shareholders, which could modify its boundaries and lead to a change in the terms of the contractual relationship.

The second type of grey zone lies in the vagueness of the boundary that separates the professional autonomy and dependence of the connected workers with regards to the requirements of the platforms (Prassl and Risak, 2016; Todolí-Signes, 2017). Where does the workers' freedom of action in carrying out their work begin and end? In France, the Supreme Court ruling of 28 November 2018 on the “Take Eat Easy” affair has partly answered this question. The court agreed that the existence of a geolocation system and a system of bonuses and penalties were two digital management tools characteristic of a relationship of subordination between a platform and a delivery rider (on behalf of their restaurant-members) and declared that the rider's service agreement should be converted into an employment contract. However, this is a specific example and is intended more to protect the worker than to define the conditions under which these tools should be used. The vague area surrounding workers' autonomy or freedom of action does not therefore entirely disappear. While labour law allows some limitations to be placed *ex post*, it does not allow intervention *ex ante* to control the use of these digital tools and to reduce the specter of contentious work situations arising. Workers will therefore never have full and unqualified freedom of action because it will always be marred by uncertainty or restrictions. From this perspective, there is an entire group of grey zones associated with the digital work environment, which, in the field, replace hierarchical surveillance and control techniques with monitoring techniques that are more or less moderate, midway between information and manipulation (see next section).

The third type of grey zone lies in the difficulty in distinguishing between the positive and negative externalities that can be seen on both sides of the platform market. The classic case often cited as an example is that of the online newspaper where the subscription is almost free or even completely free in order to increase readership. In turn, the rising number of readers attracts advertisers and increases the price of advertising space and hence cash flow for the newspaper. When looked at in this light, activity of the platform as intermediary would appear to be the reason for added value or positive pecuniary externalities. But there are also negative (non-pecuniary) externalities generated by both sides of the market and are not related to price structure but to the social cost of running the platforms (Brishen, 2017). In the case of mobility platforms, we note the impact of the Uber drivers' activity on the environment (traffic jams, pollution, etc.) or the impact of Airbnb on the hotel industry, the availability of housing, and the gentrification of cities. More broadly, the users who benefit from the lower transaction costs on platforms are often high-income and educated people. This is why the impact of platforms in terms of inequalities is significant. In addition, people significantly increase their consumption *via* a “rebound effect,” which contributes to an increase in their carbon footprint. In sum, beyond the immediate direct effects of time savings and lower transaction costs, the indirect, economic, social, and environmental effects are many and largely unknown (Frenken and Schor, 2017). This results in a problem of identification and management of these grey zones linked to interdependencies (“cross network externalities”) generated by the activity of the platform and includes a strong spatial dimension (Duranton, 1997).

In all, the grey zones can be considered as markers of the functioning of digital platforms whose impact on the activity, behaviours, and choices of users is far from neutral. In practice, the grey zones are a place of decoherence (Bureau and Dieuaide, 2018) of which the most visible sign is the distancing of wage earners' institutions (employment law and social protection). Yet the grey zones are not empty places where anarchy and chaos reign (Minassian, *op. cit*.). This is an area populated by a variety of “figures” (Azaïs, 2019b), both professional and non-professional, who work, discuss, and interact. The grey zones can therefore be zones of conflict, withdrawal, or closure, or conversely they are zones of cooperation or social innovation. In all cases, they outline a public space that is non-regulated as it is dominated by the effects of socio-spatial networks, which are cumulative and fairly stable and whose spread raises the delicate and complex problem of the non-market regulation of platform activity.

## Employment Grey Zone and New Power of Management

Digital platforms are architectures whose functioning profoundly disrupts the way in which workers' activity is organised and managed. The break with the Fordist model of production organisation is clear and unequivocal at this level. The notion of a platform ruins any conception of employment as a “place in the organisation,” owing to the lack of any organisational attachment: the workplace is no longer physically circumscribed or even geographically located; similarly, the worker's professional identity is no longer linked to the characteristics of the workplace (Huws, 2014).

In order to work, simply open an account on the platform *via* a smartphone and a dedicated application. This is a major difference from the employment contract, the execution of which by the signatory parties is based on a common will and a mutual commitment. On working platforms at least, no counterpart relationship of the “subordination for protection” type is possible. The opening of an account is a “self-declaration”: the connected worker unilaterally commits himself by becoming a member-person of the platform and declares his availability to work at any time. In summary, and to use the terms of Supiot, the allegiance relationship follows the subordination relationship (Supiot, 2015).

These changes are significant in terms of grey area analysis. Whereas under Fordism the employment relationship appears as an “effect of the employment contract,” in platform capitalism, the employment contract is replaced by a digital attachment. On the one hand, this digital attachment frees the worker from the centralised power arrangements of the hierarchical company. On the other hand, it makes the Uber driver or Deliveroo cyclist a *Homo Connecticus*, a connected worker but available and free to respond to the service or mission offers communicated by the platform. In such a context, the information collected, organised, and disseminated by the platforms is inseparable from the interpretation and decisions taken by connected workers to define and organise their actions. This is why the power to coordinate and manage platform information is also a power to manage behaviour and conduct (Deng and Joshi, 2016).

As a result, the digitisation of the worker's connection to platforms masks an opaque and deeply asymmetrical power relationship. This asymmetry is the focus of all the attention of the employers (Irani, 2015; Möhlmann and Zalmanson, 2017). It is the subject of a quasi-continual strategic reflection as to how to influence or guide the behaviour of connected workers. Taking the notion of notification as an example, the following paragraph gives an example of these “technologies of the mind” and analyses the intimate springs. A second paragraph complements these developments by emphasising a relatively unnoticed dimension in the debates, namely, the uncontrolled or undesirable effects of these techniques on the workers themselves and the different environments with which they interact.

### Notifications and Digital Governance of Labour Relations: Between Dependence and Manipulation of Connected Workers

For the connected (self-employed) worker, the working environment is summed up by the various features and applications downloaded to his or her personal mobile phone. These functions and applications are interfaces through which he is informed of the work proposals submitted to the platform. But these functions and applications are also integral components of a digital architecture placed directly under the control of platform managers. From this point of view, the instrumental and commercial rationality of management dominates with all its height and penetrates to the deepest level of the worker's cognitive processes (Fumagalli et al., 2018).

At the level of working platforms, one of the main instruments of this cognitive rationalisation is the “notification” (written or oral), even called the “nudge” (*coup de pouce* in French). We define a nudge as digital information sent to mobile phone screens or any other medium. The nudge is a “decision support” tool, as conceived by Thaler and Sunstein (2003), economist and lawyer, respectively. This tool has been used by D. Kahneman, Nobel Prize winner and a leader in the field of behavioural economics. There are many examples of nudges: the fly etched into the porcelain at the bottom of a urinal in the toilets at Amsterdam Airport, the automatic opening of a savings plan for American employees to increase the US savings rate, the marking on the ground of the words “look right” or “look left” in the streets of London to prevent accidents to tourists, or the marking on the ground of the Uber logo in several Brazilian airports to make it clear to the passenger who has just disembarked where he must go as soon as he reaches the central hall of the airport! From a more theoretical point of view, a nudge aims to correct decisions considered irrational, to fight against passivity and inertia in habits of all kinds, and to choose the “right” default options. Thaler and Sunstein use the terms “libertarian paternalism” to describe these practices, which they consider do not prohibit anything and do not restrict anyone's options (Thaler and Sunstein, 2003).

In the hands of platform managers, however, experience shows that these soft techniques for “staging” people's decisions have been recovered to serve very different objectives, such as the development and growth of corporate profits. Uber is an exemplary case in this respect. In a summary article on management practices at Guillaud (2017) identifies three main categories of nudges developed and distributed to his drivers in the debates:

- nudges encouraging people to “work harder and harder,” such as sending shopping proposals before the drivers have finished the ones in class;
- nudges to overcome “earnings loss aversions,” by informing them about high-demand areas that drivers could respond to;
- nudges seeking to “develop involvement, pleasure or play at work” by setting up a bonus system for achieving objectives defined daily by the drivers themselves.

The use of nudges by Uber management is obviously at the border between information and manipulation. The ambiguity is all the more obvious because, in return, Uber mobilises the subjectivity of “its” drivers, by playing on the lure of profit. In a masterful reversal of libertarian philosophical doctrine, Uber's research director's response to his detractors—“no one is obliged to do anything” (quoted by Guillaud)—is symptomatic of management's indifference to the drivers' working conditions, which in itself is not paradoxical because no relationship of subordination is established *a priori*. The nudges are violently denounced as denying any option for drivers to say “no” to notifications sent by the platform. Also, the absence of safeguards deprives them of any autonomy, which leads a certain number of them to work in conditions that are close to exhaustion, conditions that are contrary to their personal interest—if not that of earning more—and to that of their clients.

Thus, the nudges illustrate to a real innovation in managerial techniques for controlling the activity of connected workers. There is no physical pressure on the “bodies” as in the case of companies in the Taylorised industrial sector where work intensity is central; nor is there any need to contract workers' objectives through monetary incentives. Because of their “confinement” in a digital relationship from which workers cannot escape without disconnecting and losing their jobs, the object of control, as Benavent points out, “is no longer performance, behaviour, the sharing of common values, but the information that makes it possible to act” (Benavent, 2016, p. 30) and we will add, intelligent information, sent “to the right place and at the right time.” From this perspective, it is possible to speak of “digital Taylorism.”

In other words, the control of information systems appears to be the cornerstone of digital governance, which tends to modulate workers' ability to act by directly, relying on their full and complete availability, owing to their situation of “digital dependence” on the platform (Deleuze, 1992). The downside of this mode of governance is that it is blind to the reality of the world in which and through which workers operate. This reality is systematically obscured; it can even be perceived as an obstacle by managers who only have eyes for maximising the volume of commitments they organise on the platform and the payments that result from them.

However, this attitude finds its stumbling block in territories that appear to be places of resistance. These territories, whatever the type of administrative division or size, seem to be the only places where resistance to platforms can be expressed, with disputes from users or customers that may have a certain desire to consume, live, or produce in a sustainable way (Schor and Wengronowitz, 2017). Territories, and more generally metropolitan areas, are the infrastructure for hosting platforms. They refer not only to the world experienced by workers in the exercise of their activity but also to their socio-professional environment, that is, to all economic and non-economic actors who, directly or indirectly, individually or collectively, are the means and stakeholders who work alongside them.

### Out-of-Control and Adverse Effects Generated by Platforms

As mentioned in the *Introduction* to this article, digital technologies contain virtualities that allow them to a large extent to escape the regulations imposed by tax law, labour law, and competition law. But this ability to escape legal norms, particularly labour law, does not eliminate the various points of contact between these workspaces and the territories. Whether it is virtual space available on platform servers by means of terminals (case of Upwork), urban space such as the road network used by drivers (case of Uber), or public or domestic spaces such as stations, airports, or homes for the exercise of micro-tasks, for example (case of ATM), these workspaces are very real, physically anchored in the host territories (Orlikowski, 2007). Notably, this anchoring poses a problem if the conditions for organising and managing the resources consumed, the resources mobilised, and even the space occupied locally by workers in the very process of their activities are not (sufficiently) regulated.

The exemplary case is, here again, that of Uber drivers whose driving can constitute a danger in other public spaces if no measures are decided to ensure the safety or health of the inhabitants (pollution standards, traffic schedules and directions, speed limits, etc.). In San Francisco, for example, a team of researchers found that instead of reducing traffic jams, TNCs such as Uber and Lyft are helping to increase traffic jams. They explain that “between 2010 and 2016, the number of hours of vehicle delay during the week increased by 62% compared to 22% in a hypothetical 2016 scenario without TNCs.” Nevertheless, “the results show some substitution between TNCs and other car trips, but that most TNC trips are adding new cars to the road” (Erhardt et al., 2019, p. 10). According to the authors, municipalities face a new problem as TNCs are still growing and force local authorities “to integrate TNCs into the existing transport system” (Erhardt et al., 2019, p. 1). The researchers also point out that a large proportion of the kilometers travelled correspond to empty trips.

In other words, the functioning of the platforms is a potential source of social and collective disruption of all kinds locally. This is why it is understandable that the employment relationship, dis-institutionalised on the one hand by digitisation, is being reinvested on the other hand by a requirement to regulate professional practices, taking into account the externalities of workers' activity on the different environments that surround them or with which they interact:

- The management of externalities does not aim to protect the worker's person from the risks associated with the exercise of his profession (illness and accident) or the probability of losing his job (unemployment insurance). Managing externalities requires the involvement of all stakeholders. In the case of Uber, there are many actors: municipalities concerned with combating pollution or promoting safe mobility; driver collectives wishing to improve their remuneration and working conditions; professional unions wishing to strongly supervise the TNC profession; and chambers of commerce and industry involved in the provision of driver training, NGOs, and consumer associations concerned with the management ethics of platform managers, the quality of service, or the good morality of drivers.
- This regulation does not aim to develop a general and collective framework for the protection of all connected workers, as there is for salaried workers. The very opposite is the case. For example, by regulating the activity of Uber drivers, this regulation protects the population as a whole, active and inactive, living in an area or locality, from the effects of the inadequacy or even absence of regulation of the Uber drivers' professional activity. This regulation of the activity of connected workers can be considered as a response of local stakeholders to the disruptive power of platform managers in their function and position as third party (Collier et al., 2018). It aims to frame the reticular and sprawling dimensions of this form of power “in the field” through the mobilisation of institutional actors, what Courlet and Pecqueur call intermediation institutions (Courlet and Pecqueur, 2013). These institutions act or function locally as counter-powers, sometimes as delegated representatives of workers' interests (cf. the status of *superrogates* of alter-labour organisations in the United States, cf. Collier et al., 2017) and sometimes as defenders of broader and general interests with the aim of re-integrating the digital environment into society. Through the action of these institutions, a struggle is therefore emerging around the establishment of rules to control how platforms make use of public space, resources, and infrastructure, sometimes to the detriment of the interests of other local working and living communities. The reconquest of protection requires a struggle to impose more democracy in the definition and organisation of access to these appropriable resources, which are therefore understood as common goods for all.

To conclude this part, one would be tempted to equate the management of the externalities generated by the working platforms with the development of regulations, observed here and there, even occasionally and partially. This rapprochement highlights a process that is probably irreversible, of re-establishing the employment relationship around local (metropolitan), collective, and plural norms regulating the working conditions and context of connected workers. This process would be accompanied by the establishment of a conventional legal system that is relatively autonomous from labour law and the law, thus accrediting the thesis of the emergence of a legal pluralism (Coutu et al., 2013). At least, it can also be observed that this “remediation” is being carried out by new actors within the framework of an expanded, open, and multi-scalar public sphere by construction (Azaïs et al., 2017). So would this new standard, built on a practical territorial foundation for organising and managing the activity of platform workers, herald a co-management of the employment relationship?

## Discussion

After these few developments, we would like to return to the heuristic interest of the notion of the grey zone. Several insights can be drawn from this study.

The notion of the grey zone starts from the observation of a lasting “lack of coherence” (*decoherence* in French) between the institutions in place and the practices or behaviours they are supposed to regulate (Bureau and Dieuaide, 2018). This “decoherence” means a loss of effectiveness of both the institutions and the legal instruments available. It also means the existence of “nonstandard” social regulation, directly driven by actors or communities acting, intentionally or unintentionally, “without or outside” the established rules. The notion of a grey zone is not a catch-all category. Although it testifies to the “existence of a multidimensional crisis” (Minassian, 2018, p. 35) in existing social regulation, it is also marked by a number of perfectly identifiable characteristics, as we have tried to show. The notion of a grey zone thus makes it possible to focus on the nature and play of the extra-legal forces from which a social regulation order originates and whose dynamics coexist, overflow, or extend the instituted space of legal regulation.

The notion of “employment grey zone” is closely linked to the emergence of new professional figures. The presence of these figures can be explained to a large extent by the disruptive nature of the digital revolution (Valenduc and Vendramin, 2017). Digital platforms are learning machines that support many cognitive tasks (diagnosis, monitoring, forecasting, translation, etc.). Not only do they eliminate routine jobs, but they also encourage the development of polarisation in the labour market between skilled and unskilled workers and expose the latter to global competition. From these upheavals has come a multiplicity of professional situations and figures, more or less stable and identifiable over time. The shift of status toward precariousness, positioning on professions with an uncertain future or on upward trajectories, career development scenarios, and professional identities are never clearly defined in advance. This is why, at the heart of this grey zone, platform workers form a population of “emerging figures,” which are difficult to identify. Without being exhaustive, this population can be classified into three main categories (Azaïs, 2019b): declining figures, described as emerging as they are part of an involutionary process; intermediate figures, located in an in-between area where the short-term future is impossible to predict; and ascending figures, which provide new opportunities.

- Declining figures are the most vulnerable workers who demand more protection and apply to the courts for employee status. As they cannot find a steady job, they are compelled to use digital platforms in order to survive. More and more, TNC drivers and bicycle riders are finding themselves in such a situation. More broadly, these are workers whose work status has deteriorated and whose autonomy, pay, rights, and protection have been reduced.
- The intermediate figure corresponds to a period of transition or stagnation for the individual waiting for a more suitable job. They are individuals on stand-by, young people, women, who temporarily accept an internship or precarious status in the hope of obtaining a stable and properly paid job later. In France, this is the case for some young people in the suburbs who may have imagined that a job as an Uber driver or a bicycle rider (Jan, 2018) could allow them to reach a higher status or social position. For these young people, this was the first time they were socially recognised (Courrier international, 2016). In this category, we also find a significant proportion of women who are often the first to be threatened by the shift of jobs to digital occupations (Vendramin, 2011).
- Third, emerging figures refers to “knowledge workers,” defined as “mastering a significant part of cognitive knowledge: knowing how to master some basic transversal cognitive skills and mobile technologies, how to understand and report written and digitally transmitted instructions and, on this basis, how to relate to others, how to cooperate actively and interact” (Armano and Murgia, 2019, p. 282). We find, among this third type of emerging figure, engineers or highly skilled and very mobile workers, who will not hesitate to leave a start-up where they are already well paid to join a new one, where they will receive a better salary.

The time dimension is central to the analysis, owing to the non-permanent and shifting nature of individuals in the labour market. This typology aims to look beyond dualism, which is still significant in analyses of the current change in the employment relationship and the categories that define it. This typology also makes it possible to take into account many studies conducted on the informal sector in countries of the Global South, describing complex labour markets that cannot be confined to a binary formal vs. informal interpretation. This form of analysis, which has now been exported to the North, defines the dynamics and plurality of the forms of work and employment that can be found there. Finally, all three types of emerging figure are the expression of the multiplicity of forms of work; they also reflect people's subjectivity and their collective and personal involvement (Armano and Murgia, 2017).

There is no clear-cut demarcation between these three figures, and a single situation may illustrate two or even three types of emerging figure, as everything depends on people's lived experience and their individual and collective customs. The same situation can be lived subjectively in completely opposite ways and can be analysed as corroborating the hypothesis of generalised insecurity or as a deliberate choice on the part of individuals waiting (or not) to integrate the labour market differently. Both interpretations are possible.

The notion of grey zone provides the means to draft a framework of analysis that does not separate connected workers from the context and particular conditions in which they carry out their activities. Employment and work are thus captured in an “in-between space” (Cattaruzza, 2012), a lawless zone that does not oppose wage labour and self-employment but places them in a continuum of more or less stable forms of social relations and power. From this perspective, grey zones must be considered as open spaces, crossed by multi-actor and multidirectional dynamics. This perspective is close to the work developed by Streeck and Thelen (2005), who have established a very precise typology of the forms of institutional change centred on the actors (displacement or evolution of rules; superposition or addition of new rules; drift or *laissez-faire*; conversion or reinterpretation of rules; exhaustion or gradual rupture). In our approach, we have paid particular attention to territories, both as a support for digital infrastructures and as a “host country” for the (negative) externalities generated by digital platforms. Territories are also institutional actors concerned to guarantee the quality and access to all of the resources available within the (public) space of the division of labour. Although it remains to be demonstrated, this first overview highlights the importance of territories as important actors in the definition and implementation of a possible trajectory for the reintermediation of the employment and labour relationship. They are both carriers and pilots of a new space–time reference system in the organisation and management of work activities, and the integration of territories into the analysis is from this point of view a fruitful entry into studying the new forms of codification of the subordination link specific to digital environments.

The notion of the grey zone has emerged as a space of social relations and work immersed in the City. It is therefore a space of a directly political nature inhabited by disparate figures (emerging and non-emerging) with diverging strategies and interests. A grey zone can therefore be a zone of conflict, withdrawal, or closure, or conversely a zone of social cooperation or innovation. The notion of an emerging figure put forward in our approach responds to this concern to characterise this population more precisely in these very particular digital environments. This reflection is still in its infancy, but more broadly, we believe that the notion of the emerging figure is a very useful tool to understand how and in what form politics emerges in these “off-camera” regulations. This notion also helps to shed light on the reasons why these forces block or, on the contrary, push for institutional change. At the level of analysis, it would then be a question of identifying and understanding the emergence of new professional figures through the new problems whose work and conditions of practice are conducive to them (intervention of ecological themes, emergence of civil society actors, new forms of struggle, emergence of new trade union practices, organising, etc.). This knowledge of the field could, for example, support the idea that the notion of digital worker refers less to professions, qualifications, and new skills acquired in the digital age than to the range of rights that characterise the conditions of use or access to these tools. The notion of a grey zone would reflect a tension in the search for a socio-political balance between the exercise of work guided by necessity (working to earn a living) and work that promotes emancipation and/or freedom (cf. Marx's notion of “free activity”).

## Data Availability Statement

All datasets generated for this study are included in the article/supplementary material.

## Author Contributions

All authors listed have made a substantial, direct and intellectual contribution to the work, and approved it for publication.

**JEL: J23, J53, J8, L86, M54**

### Conflict of Interest

The authors declare that the research was conducted in the absence of any commercial or financial relationships that could be construed as a potential conflict of interest.

## Footnotes

^1^As part of this contribution, we will focus on labour platforms, understood in a broad sense as service operators. Uber drivers, Deliveroo couriers, Amazon Mechanical Turk (AMT) “taskers,” self-employed workers connected to Upwork fall into this category. For Eurofound (2018), five criteria are used to define a working platform: paid work is organised through online platforms; three parties are involved—the online platform, the worker, and the client; work is contracted out; jobs are broken down into tasks; and services are provided on demand. In this work, we will focus in particular on the second criterion, which refers to the function of platforms as market intermediaries.

^2^According to an Uber judgment of 20 December 2017 by the European Court of Justice (ECJ), “The service of connecting with non-professional drivers provided by Uber is part of the transport services (the connecting service is a secondary element and it is the provision of transport which is, from an economic point of view, the main element). Such a service must therefore be excluded from the scope of the freedom to provide services in general and from the Directive on services in the internal market and the Directive on electronic commerce” https://curia.europa.eu/jcms/upload/docs/application/pdf/2017-12/cp170136en.pdf, consulted on 25 July 2019.

^3^This hold that the platforms have over the data can be explained by the fact that the connection is based on a digital link that is the private property of the platform (for example, the “like” link on Facebook is the property of Facebook).

^4^In Europe, see the General Data Protection Regulation (GDPR), which came into force on 25 May 2018. This legislation deals with the processing and circulation of data in the EU by giving people whose data have been collected and processed a certain number of rights (right to access, right to correction, right to objection, right to erasure, and right to portability).

^5^As Gauron points out: “Contrary to a presentation firmly anchored in economic history, the specific feature of platforms cannot be found in technology. It lies in a change in convention compared with Fordism, the transition from an industrial convention to a market convention” (Gauron, 2017). To complement these remarks, it should be noted that the market convention is a means of coordination by the market with prices fixed during the exchange. However, it is never totally so: in the case of Uber, the price of the trip is not freely set, and nor is it based on an agreement between the customer and the driver. It is fixed by algorithms managed by the company and is exactly the same for all drivers. The diffusion of the platforms leads to the revival of long-standing criticisms of the neoclassical approach to markets, which considered the market secretary (the famous auctioneer by Walras) not as a metaphor for the invisible hand but as the embodiment of a centralised economy regulated by a planning state (Guerrien, 2006).
